# Supplementary material for: Investigating diversity in European audiences for public engagement with research: Who attends European Researchers’ Night in Ireland, the UK and Malta?
Source: PLoS One. 2021 Jul 14;16(7):e0252854. doi: 10.1371/journal.pone.0252854 (PMC8279411; doi:10.1371/journal.pone.0252854)
Supplement: Attachment — Submitted filename: PLOSONE - detailed responses to reviews (handover).docx [file pone.0252854.s001.docx]

Investigating Diversity in Public Engagement with Research: Who attends European Researchers’ Night in Ireland, UK and Malta?

Responses to PLOSONE review comments

| **Comment ID** | **Reviewer ID** | **Comment** | **How did you address the comment *(quote what you added here if relevant) - write in a way that it can be shown directly to the editors with the resubmission*** |
| --- | --- | --- | --- |
| 001 | Reviewer #1 | Authors could say more about why they propose hypothesis 2. The previous evidence shared in the paper suggests that audiences for ERN events may be more likely to have higher educational qualifications (hypothesis 1), but there is no literature cited by the authors to explain why the research team thought that ERN events would attract white participants. Citing more scholarship on the lack of racial/ethnic diversity in STEM fields would be helpful to better motivate/present hypothesis 2. | While there are no published studies of the ethnicity of ERN audiences, there has been an ongoing discussion in the published literature of the over-representation of white majority participants in public engagement and science communication events. Several of the most recent papers that have discussed racial diversity in science communication are now referenced in the introduction. |
| 002 | Reviewer #1 | While the data is sound, the data does not support the conclusions regarding hypothesis 2. The data support conclusions regarding hypothesis 1. In the analysis of hypothesis 2, the discussion of ethnic minority participants states that the ethnic diversity of the adult attendees were similar to or greater than their respective populations. However, this was not the case in the UK, so I found this to be a puzzling conclusion given the data shown in table 6. Whites were over-represented by 2.3% in the UK and Blacks were under-represented by 1.5%. | This comment suggests the phrasing in the Discussion section could be misleading. This has been rephrased as follows: The second hypothesis, that ‘like science festivals, ERN events attract an audience that over-represents white majority participants compared to the general population (and under-represent minority ethnicities)’ could not be upheld. Data to address this hypothesis were available from samples in two countries: In Ireland, there was a -15.6% underrepresentation of White participants compared to population, +13% Asian and +2.3 Black. In the UK, there was a small level of over-representation of white majority participants in line with the hypothesis (+2.3% White). Taken together, we have clear evidence of over-representation of ethnic minority groups at engagement events in one country, and a small level of over-representation of White ethnic groups in the other. This mixed bag of evidence for ethnic minority representation in public engagement with research audiences is insufficient to uphold Hypothesis 2. |
| 003 | Reviewer #1 | The authors don't share the ethnic data for the Malta event and do not explain why the ethnicity data is not reported for the Malta event. What did the study find about ethnic minority participants in Malta? Based on the incomplete data presented here, it would be more accurate to conclude that there were national differences in ethnic minority participation in ERN events. So results were inconclusive regarding hypothesis 2. | We have added an explanation in the Methods section: “In Malta, the National Statistics Office did not, at the time of this study, identify ethnicity in its national (census) survey, therefore ethnic diversity could not be benchmarked for the Maltese sample. This prevented Maltese data from contributing to assessment of Hypothesis 2.” |
| 004 | Reviewer #2 | The data in Table 1 is a little confused with regard to secondary education. It seems to include the Irish data for Senior and Junior Certificate and the remaining countries under Secondary Education. I recommend consolidating secondary education under one heading. | We have consolidated the Senior and Junior Certificate Categories under Secondary Education. This is now reflected in Table 3 (Comparison of educational attainment of respondents across all event locations) |
| 005 | Reviewer #2 | In the results section, it becomes clear that only adult attendees were surveyed. I recommend stating this point clearly in the Methods section. | The method section now notes that the survey was administered to adults. |
| 006 | Reviewer #2 | The statement in the abstract about "over-inclusion" of ethnic minorities in ERN activities is overstated: it depends very strongly on one immigrant community, Asians, in one country, Ireland. The comments in the results section are more measured and accurate. No attempt is made to discuss the significant differences in the demographics of Asian immigration to Britain and Ireland. It may be that the age and education profile of Asian immigrants in Ireland is very significantly different. | We have adjusted the phrasing in the abstract to mitigate this concern. (We believe going into ‘the significant differences in the demographics of Asian immigration to Britain and Ireland’ would be beyond the scope of the current exploratory study.) |
| 010 | Reviewer #3 | This manuscript, as it stands now, is not a true research study. It is missing a) sound hypotheses, b) comparisons to controls, c) statistical analyses, and d) correlations with and influences of major causal factors. | This is an exploratory and descriptive study, which does not attempt to make grand causal claims. This fact is now more explicit in the framing of the paper in the abstract, introduction, results and conclusion in hopes of tempering expectations. Citations have been added to methodological literature that supports the validity of the approach used and explains why it is necessary in real-world audience research contexts (Reviewer 3’s description of a ‘true research study’ does not comport with accepted research methods in the social sciences - and indeed parts of natural science, where naturalistic field studies rarely can include control groups or employ an experimental design more generally.) |
| 011 | Reviewer #3 | The rationale and evidence behind hypothesis 2 (expectations for attendance distribution between white and non-white demographics) are not provided questioning the purpose of this study. | Added Dawson 2018 to clarify rationale and evidence behind H2 for why we expected to see an issue with ethnicity. |
| 012 | Reviewer #3 | Socioeconomic and education status are conflated with each other and used interchangeably throughout the paper – the authors need to be clear about what they are studying and need to articulate the relationship between education and socioeconomic status based on evidence. | The writing has been adjusted to make it more clear how the claims about socioeconomic status connect to the evidence about educational attainment. We note that educational status is one of the most well-established dimensions of socioeconomic status: <https://www.apa.org/topics/socioeconomic-status> |
| 013 | Reviewer #3 | There is no mention of the merits of or the biases introduced via the “intercept” data collection in the demographic representation. | The implication in this question has now been addressed with this clarification: ‘Where demographic questions were potentially sensitive (e.g. regarding ethnicity or income), respondents were either given the tablets to complete their answers independently or these questions were saved for the post-visit survey.  In addition, this is partially addressed with the note about intercept surveys being standard practice in audience research: ‘..., as is standard in audience research settings to mimic random selection to the extent feasible (e.g. Jensen & Lister, 2015)’ |
| 014 | Reviewer #3 | The sample size claim is misrepresented. Out of the 2092, 317 declined to participate in their study. | We have clarified the distinction between Sample Frame and Achieved Sample Size throughout. The Abstract now reflects the latter figure. |
| 015 | Reviewer #3 | The authors fail to address the influence of the event location on the educational status and ethnic representation seen in the events. For example, events in Ireland were hosted on university campuses and thus likely attracted more educated people including immigrant researchers (contributing to more nonwhite people attending). Local demographic comparisons are lacking – for example, population demographics of the city where the event was hosted in comparison to event attendance is a comparison to be made. In a country like Ireland, there likely are huge variations in demographics between urban & rural areas thus influencing their ultimate data. | The ethnicity of the student population has been included in the Results section, below Table 6 (Comparison of ethnicity of national population and participating science festival visitors) to demonstrate a clear example, using the available data, of how the university campus demographics were closer to the general population than to the profile of the ERN audience. |
| 016 | Reviewer #3 | The authors claim that ERN event demographics mirror other science festival events, although the direct comparison of attendance with previous events is not provided. Overall, the data severely lack controls or statistical analysis to make claims. How does this specifically compare to data on other attendance data such as museum memberships, science festival attendances, outreach event attendances? Is it statistically different? | As noted in the paper, the link to science festivals is made because it is the closest existing evidence relevant to public engagement with research audiences in the published literature. The paper does not claim that ERN event demographics precisely map onto science festival results. To make the distinction clearer, we have deleted the ‘like science festivals’ in both the hypotheses. |
| 017 | Reviewer #3 | The primary source and the year of the national population data used for comparisons are not noted – this is important as the data in the paper are across many years and it is known that national demographics change with time. | The primary source and the year of the national population data used for comparisons are now noted under Methods: Data Analysis section. Further citations have been added to References. |
| 018 | Reviewer #3 | The correlation of educational status to ethnic representation is not considered or even discussed – are some ethnicities overrepresented in people with or without college degrees? | Conducted and reported the analysis for determining statistically significant relationships between the two categories of data. No statistical significance is found. We did also produce a table with corresponding descriptive statistics (n, %), but agreed it is not sufficient to include as a result. |
| 019 | Reviewer #3 | The authors make blanket conclusions about ethnicity data despite huge differences in the demographics of attendance pattern the UK vs Ireland. Moreover, ethnicity data from the country of Malta is completely missing. The authors fail to acknowledge this. | The aspect of Malta has been addressed in a previous comment by Reviewer 1 (003) |
| 020 | Reviewer #3 | The survey language is not provided in the Methods section. | The method section now notes that the survey instrument was in English. |
| 021 | Reviewer #3 | The exact type of activities and specific examples that constitute the ERN events are sparsely described and no information is provided for activity types in the UK whatsoever. | Have provided further clarification on characteristics of the UK programme of events relevant to our sampling. |
